# Comparison of the *Glaesserella parasuis* Virulence in Mice and Piglets

**DOI:** 10.3389/fvets.2021.659244

**Published:** 2021-06-24

**Authors:** Baichuan Qi, Feiyue Li, Kunpeng Chen, Wenwen Ding, Yun Xue, Yang Wang, Hongwei Wang, Ke Ding, Zhanqin Zhao

**Affiliations:** ^1^The Key Lab of Veterinary Biological Products, Henan University of Science and Technology, Luoyang, China; ^2^Henan Provincial Open Laboratory of Key Disciplines in Environmental and Animal Products Safety, Henan University of Science and Technology, Luoyang, China

**Keywords:** *Glaesserella parasuis*, serovar, virulence, mouse, piglet, correlation

## Abstract

In this study, we compared the virulence of the most common serovars of *Glaesserella parasuis* in China, serovars 4, 5, 12, and 13 (36 strains in total) in BALB/c mice and piglets. In mice, the median lethal doses (LD_50_s) of the four serovars were roughly 9.80 × 10^7^–4.60 × 10^9^ CFU, 2.10 × 10^8^–8.85 × 10^9^ CFU, 4.81 × 10^7^–7.01 × 10^9^ CFU, and 1.75 × 10^8^–8.45 × 10^8^ CFU, respectively. Serovar 13 showed the strongest virulence, followed by serovar 4, serovar 12, and serovar 5, but a significant difference in virulence was only observed between serovars 5 and 13. The virulence of strains of the same serovars differed significantly in piglets. Virulent and attenuated strains were present in all serovars, but serovar 5 was the most virulent in piglets, followed by serovars 13, 4, and 12. A significant difference in virulence was observed between serovars 5 and 4 and between serovars 5 and 12. However, the virulence of serovars 5 and 13 did not differ significantly. This comprehensive analysis of *G. parasuis* virulence in mice and piglets demonstrated that: (1) the order of virulence of the four domestic epidemic serovars (from strongest to weakest) in piglets was serovars 5, 13, 4, and 12; (2) both virulent and attenuated strains were present in all serovars, so virulence did not necessarily correlate with serovar; (3) Although *G. parasuis* was fatal in BALB/c mice, its virulence is inconsistent with that in piglets, indicating that BALB/c mice are inadequate as an alternative model of *G. parasuis* infection.

## Background

*Glaesserella parasuis* is a small Gram-negative bacillus belonging to the family Pasteurella, which causes polyserositis, arthritis, and sepsis in pigs, collectively called “Glässer's disease” (1). This is a common disease that seriously threatens the pig industry worldwide (2). In 1992, Kielstein and Rapp-Gabrielson developed a serological typing assay using an agar diffusion test that successfully categorizes *G. parasuis* into at least 15 serovars (3). Serovars 4 and 5 are most common, followed by serovars 13 and 14, and these have distinct spatial and temporal distributions (4–10). Kielstein and Rapp-Gabrielson also defined serovars 1, 5, 10, 12, 13, and 14 as virulent strains, serovars 2, 4, 8, and 15 as mesogenic strains, and serovars 3, 6, 7, 9, and 11 as attenuated strains (3). However, follow-up studies have shown that *G. parasuis* virulence does not necessarily correlate with the serovar (11). In recent years, the virulence-related genes of *G. parasuis* have been identified, but the pathogenic mechanisms of *G. parasuis* infection have not been demonstrated (12–16). Therefore, the determination of *G. parasuis* virulence still relies on infection tests in pigs.

Specific-pathogen-free (SPF) pigs (17, 18), Cesarean-derived, colostrum-deprived (CDCD) piglets (19), naturally farrowed, artificially reared (NFCD) pigs (20), and 9–10-week-old piglets negative for anti-*G. parasuis* antibodies (21, 22) have been successfully investigated. However, using piglets as a model of infection requires high standard laboratory conditions and expensive equipment. Alternative models have been explored with different strains of mice (23–26), but the BALB/c mouse is the only widely used alternative model. However, some previous studies using BALB/c mice as an alternative infection model have produced inconsistent and even contradictory results (26–29), and there has been no comparative study of the alternative BALB/c mouse model and the piglet model.

Experimental intraperitoneal infection model in BALB/c mice and piglets are used extensively in research on information for virulence and immunology of *G. parasuis* (22–26). In this study, BALB/c mice and 8–9-week-old *G. parasuis* seronegative piglets were used as the challenge models. The virulence of 36 common strains of *G. parasuis* in China was measured, and the reliability of the infection model in BALB/c mice was evaluated. Our results provide new data that extend our understanding of the potential harm posed by these *G. parasuis* strains, and should have utility in the diagnosis and prevention of Glässer's disease.

## Methods

### Strains, Media, and Reagents

Thirty-six *G. parasuis* strains, including eight strains of serovar 4, 12 strains of serovar 5, eight strains of serovar 12, and eight strains of serovar 13, were isolated, identified, and stored at the Veterinary Laboratory of Bioengineering of Henan University of Science and Technology (Table 1). Tryptose Soya Agar (TSA) and Tryptose Soya Broth (TSB) media were purchased from Becton, Dickinson and Company (Sparks, MD, USA). Newborn calf serum was purchased from Zhejiang Tianhang Biotechnology Co. Ltd. Nicotinamide adenine dinucleotide (NAD) was purchased from Sigma-Aldrich.

**Table 1:** Results of mice virulence tests on different serovars.

**Number**	**Strain**	**Serovar**	**Source**	**Isolation time**	**LD_**50**_ (CFU)**	**LgLD_**50**_**	**Geometric mean LgLD_**50**_**
1	H134	5	Hunan	2,013.11	2.10 × 10^8^	8.32	9.23
2	H109	5	Henan	2,013.09	4.03 × 10^8^	8.61	
3	H117	5	Hubei	2,016.11	5.26 × 10^8^	8.72	
4	H110	5	Henan	2,014.07	1.68 × 10^9^	9.23	
5	H126	5	Hubei	2,015.08	1.83 × 10^9^	9.26	
6	H116	5	Hubei	2,014.04	1.98 × 10^9^	9.30	
7	H102	5	Guangdong	2,015.09	2.30 × 10^9^	9.36	
8	H106	5	Henan	2,013.01	2.52 × 10^9^	9.40	
9	H122	5	Hubei	2,014.03	2.58 × 10^9^	9.41	
10	H94	5	Henan	2,017.06	3.18 × 10^9^	9.50	
11	H123	5	Hubei	2,013.01	7.90 × 10^9^	9.90	
12	H101	5	Guangdong	2,015.12	8.85 × 10^9^	9.95	
13	H26	4	Henan	2,015.11	9.80 × 10^7^	7.99	8.89
14	H57	4	Henan	2,013.01	1.13 × 10^8^	8.05	
15	H70	4	Henan	2,013.07	2.00 × 10^8^	8.30	
16	H58	4	Henan	2,017.12	5.51 × 10^8^	8.74	
17	H47	4	Henan	2,016.10	2.14 × 10^9^	9.33	
18	H44	4	Henan	2,013.03	2.82 × 10^9^	9.45	
19	H52	4	Henan	2,013.04	4.23 × 10^9^	9.63	
20	H35	4	Henan	2,014.01	4.60 × 10^9^	9.66	
21	H168	12	Henan	2,015.07	4.81 × 10^7^	7.68	8.91
22	H173	12	Henan	2,013.09	5.17 × 10^7^	7.74	
23	H170	12	Henan	2,013.08	5.55 × 10^8^	8.74	
24	H163	12	Hubei	2,017.09	8.64 × 10^8^	8.94	
25	H186	12	Henan	2,015.06	1.82 × 10^9^	9.26	
26	H180	12	Guangdong	2,013.01	2.42 × 10^9^	9.38	
27	H187	12	Henan	2,016.05	5.06 × 10^9^	9.70	
28	H157	12	Henan	2,017.06	7.01 × 10^9^	9.85	
29	H209	13	Guangdong	2,015.03	1.75 × 10^8^	8.24	8.62
30	H199	13	Henan	2,016.08	2.45 × 10^8^	8.39	
31	H204	13	Henan	2,014.11	3.40 × 10^8^	8.53	
32	H201	13	Henan	2,015.04	3.77 × 10^8^	8.58	
33	H208	13	Guangdong	2,013.12	3.96 × 10^8^	8.60	
34	H194	13	Shanghai	2,015.11	6.50 × 10^8^	8.81	
35	H210	13	Guangdong	2,013.01	7.96 × 10^8^	8.90	
36	H200	13	Henan	2,016.06	8.45 × 10^8^	8.93	

*The gray shades is used to distinguish the serotypes of the strains*.

### Animals

Female SPF BALB/c mice (6–8 weeks old) were purchased from Charles River Co., Ltd, and 8–9-week-old piglets were purchased from farmers in Yichuan County, Henan Province, China. Nasal swabs were collected from all the piglets used in this study and shown to be *G. parasuis*-negative with PCR (30). The collected sera were shown to be *G. parasuis*-antibody negative with a microplate agglutination test (MAT) (31). All animal experiments were conducted in accordance with the guidelines and with the approval of the Animal Experiment Committee of Henan University of Science and Technology (No. 20190719016).

### Mouse Virulence Test

TSA plates containing 10% newborn calf serum and 10 μg/mL NAD were inoculated with *G. parasuis* strain H134 of serovar 5 and incubated at 37°C for 24–48 h. Individual colonies were picked, purified, and cultured for 24–36 h. The cultured colonies were transferred to culture dishes containing 5–8 mL of phosphate-buffered saline (PBS; pH 7.2) with a sterile technique. The colonies were thoroughly suspended by pipetting. Three concentrations of the H134 bacterial suspension were prepared with two-fold dilution. When determining the virulence of strain H134, sixteen BALB/c mice were divided into four groups (four mice in each group). The original H134 bacterial suspension and the three dilutions were injected intraperitoneally into the mice of each group (0.2 mL per mouse). The H134 bacteria in the original suspension were counted to determine the actual numbers inoculated.

The remaining *G. parasuis* strains were cultured and used to challenge the mice in the same way. One PBS blank control was established for each batch. The incidence of disease and death were observed and recorded. The experiment was terminated 14 d post-challenge; survivors being killed with an intravenous overdose of sodium pentobarbital. Autopsies were performed immediately on the dead mice to record the numbers of lesions. Cardiovascular and pulmonary blood samples were collected and the bacteria in the samples identified. The median lethal dose (LD_50_) of *G. parasuis* in the mice was then calculated with the Reed-Muench method (32).

### Piglet Virulence Test

*G. parasuis* strain H134 was cultured in medium plates as described above. Then a single colony of strain H134 was picked and cultured in TSB medium containing newborn calf serum (10%) and NAD (10 μg/mL), with shaking at 180 rpm at 37°C for 12–16 h. The culture solution was used as the stock solution and diluted 1:100, transferred to TSB medium, and cultured with shaking at 180 rpm at 37°C for 10–14 h. The H134 bacteria were harvested when their concentration was maximum, and the optical density at a wavelength of 600 nm (OD600) as well as growth curve of each strain were previously detailed (33) (Supplementary Figure 2). The cultured H134 bacterial solution (3 mL) was injected into the abdominal cavities of five piglets housed in isolation in pens with a concrete floor after they had been fasted for 4 h. The number of H134 bacteria in the original solution was counted to determine the actual number of bacteria inoculated (34).

The remaining *G. parasuis* strains were cultured and used to challenge the pigelts in the same way. Thirty-six *G. parasuis* strains were tested in batches, and five piglets inoculated with sterile PBS were included in each batch as the controls. Disease onset and death were observed and recorded for 14 days. Autopsies were performed immediately on the deceased piglets, and bacteria were isolated from the heart blood and lungs and identified. After observation, all the survivors were killed with an intravenous overdose of sodium pentobarbital and dissected to check for lesions. The *G. parasuis* strains were quantitatively scored according to the morbidity and death of the piglets (Table 2).

**Table 2:** Piglet virulence test results of different serovars.

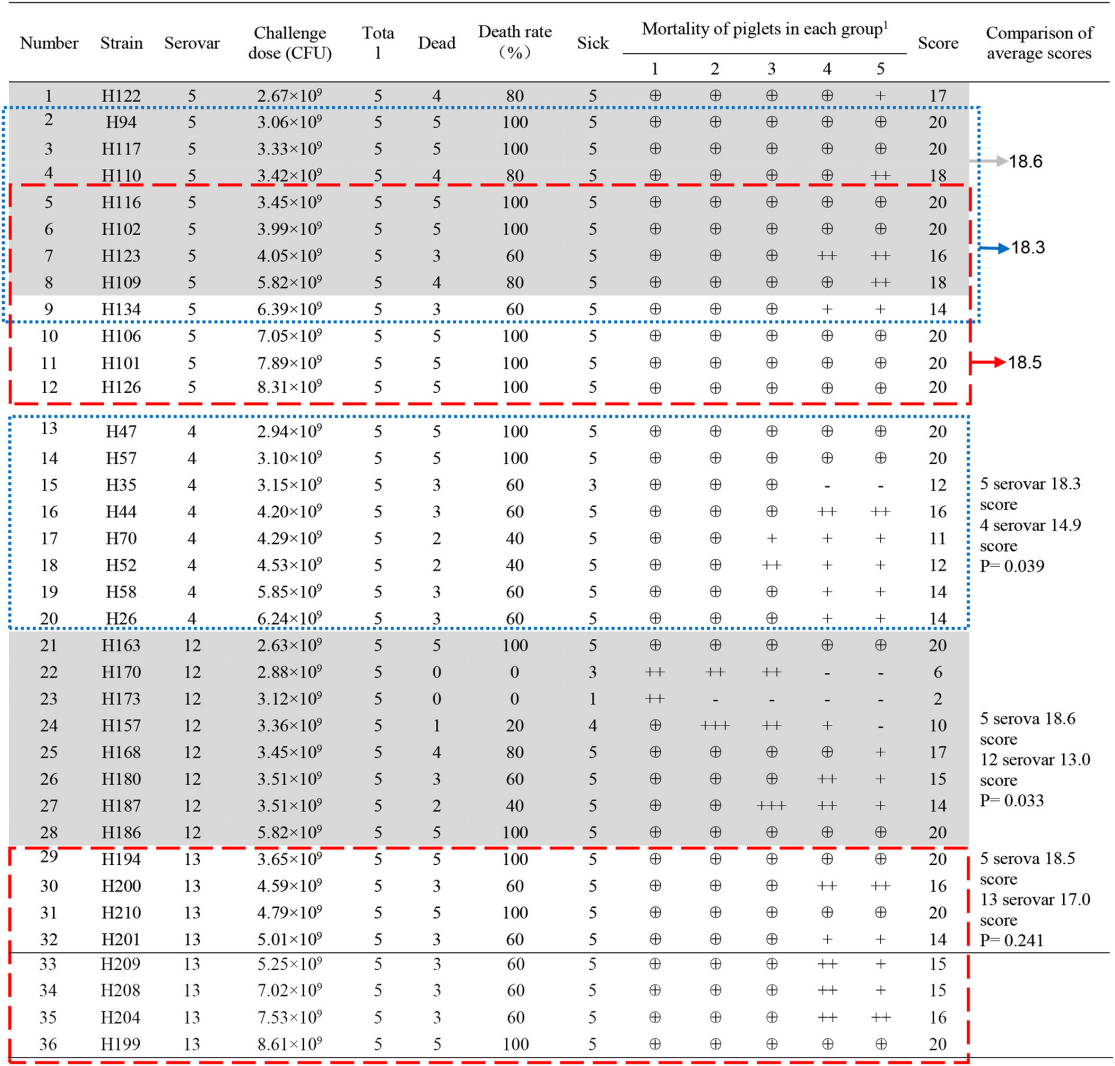

*The meaning of superscript “1” is “⊕”: death, 4 points; “+++”: severe, 3 points; “++”: moderate, 2 points; “+”: slight, 1 points; “–”: normal, 0 points*.

*“Blue frame” “Red frame” “Shaded” indicate the corresponding strains of serovar 5 when compared with serovars 4, 13, 12, respectively*.

*There are 12 strains of serovar 5, 8 strains of serovars 4, 12, and 13, respectively. The strains of serovars 4, 12, and 13 were compared with those of serovar 5 (eight consecutive strains with similar amounts of challenge bacteria). When the average virulence score was used as a reference, a large value indicated strong virulence, whereas a small value indicated weak virulence*.

### Data Analysis

The logarithm of median lethal dose (LgLD_50_) value is used to indicate virulence (a higher value indicates weaker virulence). ANOVA was used to compare the LgLD_50_ values. *P* < 0.05 was deemed to indicate a significant difference.

The comprehensive virulence of various serovars in piglets was compared after normalization by sample size and the amount of bacteria used in the challenge. There were 12 strains of serotype 5 and 8 strains of serotype 4, serotype 5 and serotype 12 respectively. “Blue frame,” “Red frame,” and “Shaded” indicate the corresponding strains of serovar 5 when compared with serovars 4, 13, 12, respectively. The strains of serovars 4, 12, and 13 were compared with those of serovar 5 (eight consecutive strains with similar amounts of challenge bacteria; Table 2). When the average virulence score was used as a reference, a large value indicated strong virulence, whereas a small value indicated weak virulence. Student's *t*-test was used to evaluate the virulence scores of each type of strain. *P* < 0.05 indicated a significant difference.

SPSS 25.0 was used for all data analyses.

## Results

### Virulence of Epidemic Serovars in Mice

After the mice were infected with strains of four *G. parasuis* serovars, they displayed listlessness and disordered hair, and ceased or reduced their consumption of food. All the dead mice died within the first 3 days of challenge, whereas the surviving mice quickly recovered both their mental state and food intake. The mice in the blank control group showed no symptoms or deaths. *G. parasuis* was isolated from the blood samples collected from the heart and lungs of the dead mice during autopsy. All the mice infected with *G. parasuis* showed anorexia, psychiatric disorders, astasia, convulsion, cyanosis with visible mucous membrane. Hemorrhage and congestion were the main pathological features in the heart, liver, and other organs (Supplementary Figure 1).

The LD_50_ values of the strains were calculated with the Reed-Muench method, and the results (Table 1) showed that the LD_50_s of eight strains of *G. parasuis* of serovar 13 were between 1.75 × 10^8^ and 8.45 × 10^8^ CFU. The LD_50_ values of the three other serovars varied, and could be divided into two intervals. In the eight *G. parasuis* strains of serovar 4, the LD_50_s of four strains ranged between 9.80 × 10^7^ and 5.51 × 10^8^ CFU, whereas those of the other four strains ranged between 2.14 × 10^9^ and 4.60 × 10^9^ CFU. In the eight strains of serovar 12, the LD_50_s of four strains ranged between 4.81 × 10^7^ and 8.64 × 10^8^ CFU, whereas those of the other four strains ranged between 1.58 × 10^9^ and 7.01 × 10^9^ CFU. In the 12 *G. parasuis* strains of serovar 5, the LD_50_s of three strains ranged between 2.10 × 10^8^ and 5.26 × 10^8^ CFU, whereas those of the other nine strains ranged between 1.68 × 10^9^ and 8.85 × 10^9^ CFU.

**Figure 1:**
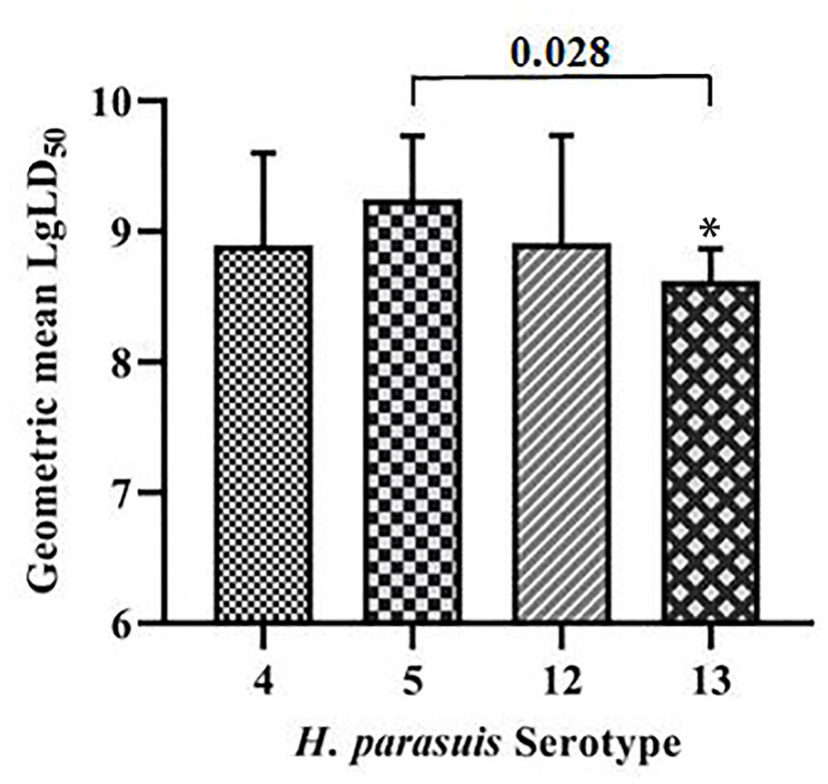
Comparative results of mouse virulence tests for the strains of each serovar. *P < 0.05 and **P < 0.01, compared with *G. parasuis* serovar 5.

The geometric means of the LgLD_50_ values were examined to analyze the virulence of each serovar in mice (35, 36) (Table 1). Serovar 13 was most virulent, followed by serovars 4, 12, and 5, which displayed the weakest virulence. However, ANOVA indicated that only the virulence of serovars 5 and 13 differed significantly (*P* < 0.05), and no significant differences were observed in the virulence of the other strains (*P* > 0.05; Figure 1).

### Virulence of Epidemic Serovars in Piglets

**Figure 2:**
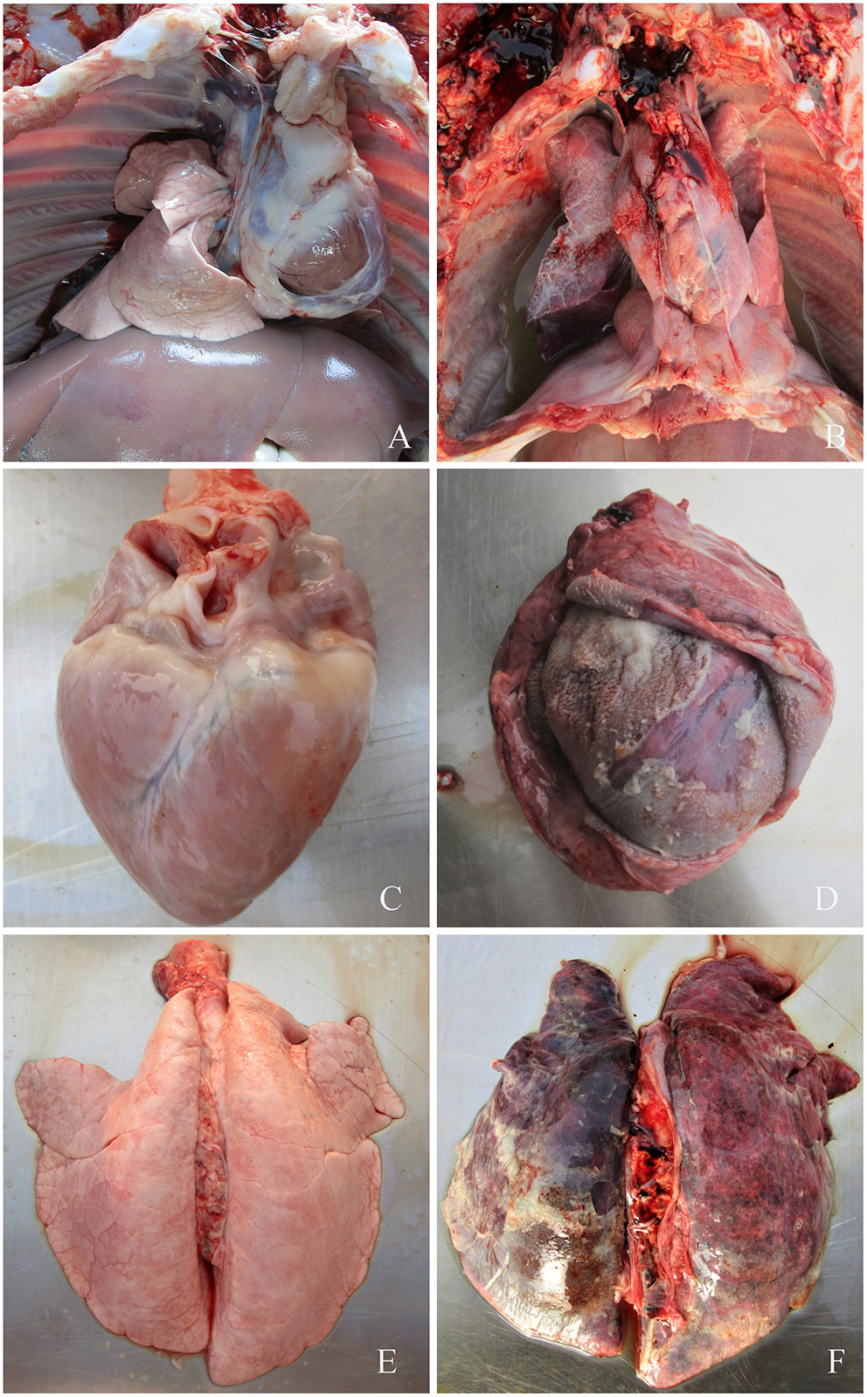
**(A)** Symptoms of piglets infected with *G. parasuis*. **(A,C,E)** control group; **(B)** pleural effusion; **(D)** pericardial thickening, typical “fluff heart”; **(F)** pulmonary congestion, bleeding, necrosis.

**Figure 3:**
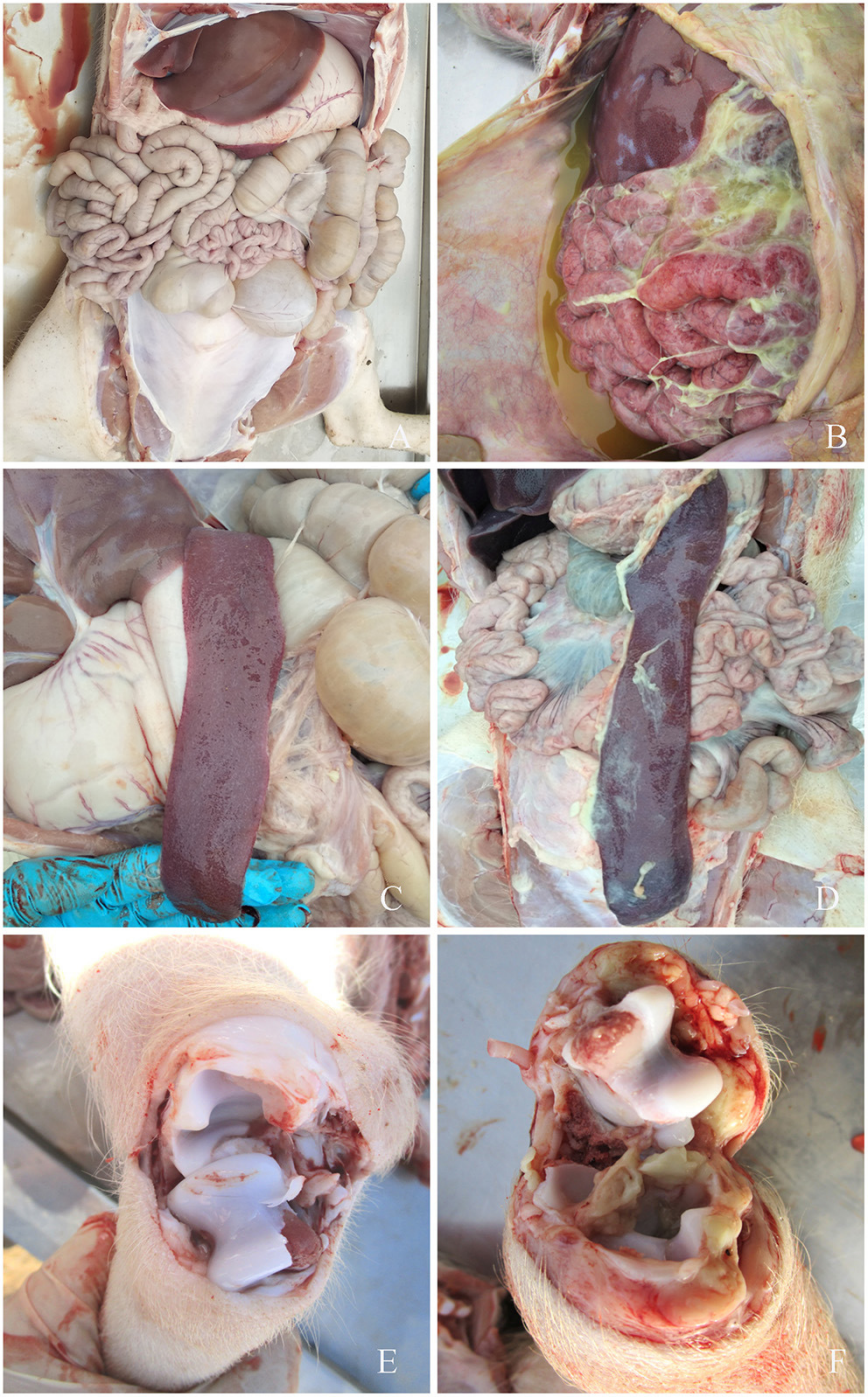
Symptoms of piglets infected with *G. parasuis*. **(A,C,E)** control group; **(B)** peritoneal fibrous exudate, intestinal wall congestion, adhesions; **(D)** spleen edge infarction, covered with fibrous pseudomembrane; **(F)** joints effusion.

None of the piglets in the control group manifested symptoms. However, symptoms typical of *G. parasuis* disease appeared in the piglets infected with each serovar strain, mainly manifesting as increased body temperature (>40.5°C), depression, loss or diminution of appetite, difficulty in breathing, skin cyanosis, joint swelling, and claudication. In a later stage, disordered hair, weight loss, lying on the ground, and death were observed. The dead piglets were immediately necropsied, and severe serous, fibrinous exudations and sepsis were observed. *G. parasuis* was isolated from the heart, blood, and lungs of the dead piglets (Figures 2, 3).

The detailed results are shown in Table 2. Seven strains of *G. parasuis* serovar 5 caused the death of all piglets (7/12). The remaining five strains caused a death rate of 60% or above. Three strains of *G. parasuis* serovar 13 (3/8) caused death in all the piglets, and the remaining five strains resulted in a death rate of 60%. Two strains of *G. parasuis* serovar 4 caused the death of all piglets (2/8), four strains resulted in a death rate of 60%, and the remaining two strains caused a death rate of only 40%. Serovar 12 showed the weakest overall virulence (Table 2).

**Figure 4:**
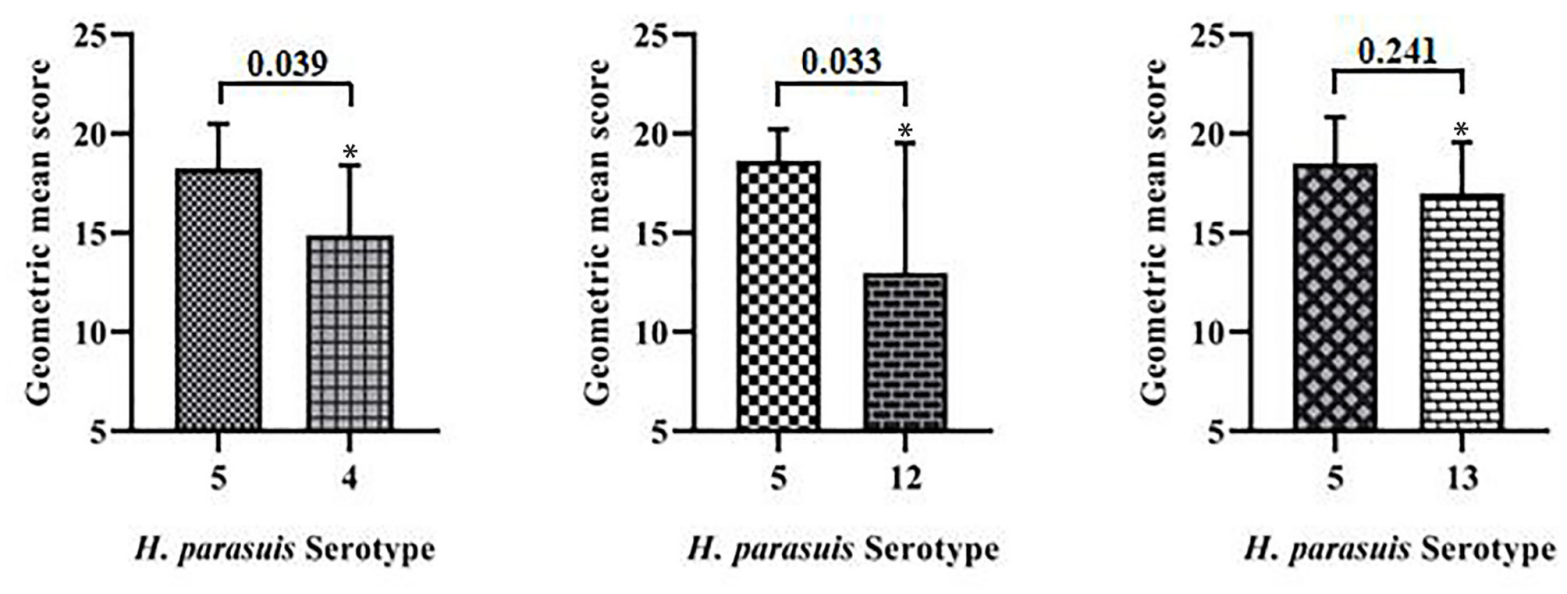
Comparison of the comprehensive virulence of serovars 4, 12, and 13 with that of serovar 5 in piglets. *P < 0.05 and **P < 0.01, compared with *G. parasuis* serovars 5.

The comprehensive virulence of three serovars was compared with that of serovar 5. The comprehensive scores were calculated according to the severity of the onset of symptoms (Table 2) and the results were analyzed statistically. The comprehensive scores for serovar 5 and 4 were 18.3 and 14.9, respectively, which differed significantly (*P* = 0.039); the comprehensive scores for serovars 5 and 12 were 18.6 and 13.0, respectively, which differed significantly (*P* = 0.033); the comprehensive scores for serovars 5 and 13 were 18.5 and 17.0, respectively, which differed significantly (*P* = 0.241). These results showed that serovar 5 had the strongest comprehensive virulence, followed by serovar 13 and 4, and that serovar 12 had the weakest comprehensive virulence. Moreover, the comprehensive virulence of serovar 5 was significantly stronger than that of serovar 4 (*P* = 0.039) and 12 (*P* = 0.033; Figure 4).

### Comparison of *G. parasuis* Virulence in Mice and Piglets

The virulence of the various *G. parasuis* serovars differed significantly in mice and piglets. Furthermore, the virulence of different strains of the same serovar also varied significantly. However, the comprehensive virulence of each serovar was inconsistent in mice and piglets. Serovar 13 showed the strongest comprehensive virulence in mice, followed by serovar 4, 12, and 5, and the comprehensive virulence of serovar 5 was significantly weaker than that of serovar 13 (*P* < 0.05). However, *G. parasuis* serovar 5 showed the strongest virulence in piglets, followed by serovar 13, 4, and 12. Furthermore, the comprehensive virulence of serovar 5 was significantly stronger than that of serovar 4 and 12 in piglets (*P* < 0.05). The virulence of 36 strains in mice and piglets was compared, and six strains were consistent. Furthermore, one strain showing either strong, medium, or weak virulence in piglets was selected from each serovar and their virulence (LD_50_) in mice examined to test the consistency of the results (Table 3). However, although *G. parasuis* could kill mice, the virulence of its various serovars was inconsistent with that in piglets.

**Table 3:** Comparative analysis of mice and piglet virulence tests.

**Serovar**	**Strain^**a**^**	**Challenge dose (CFU)**	**Death rate (%)**	**Virulence of pigs^**b**^**	**Mice LD_**50**_**	**Virulence of mice^**b**^**	**Virulence consistency^**c**^**
4	H47	2.94 × 10^9^	100	H	2.14 × 10^9^	M	N
	H44	4.20 × 10^9^	60	M	2.82 × 10^9^	W	N
	H70	4.29 × 10^9^	40	W	2.00 × 10^8^	H	N
5	H94	3.06 × 10^9^	100	H	3.18 × 10^9^	M	N
	H122	2.67 × 10^9^	60	M	7.90 × 10^9^	W	N
	H134	6.39 × 10^9^	60	W	8.10 × 10^7^	H	N
12	H163	2.63 × 10^9^	100	H	8.64 × 10^8^	M	N
	H180	3.51 × 10^9^	60	M	2.42 × 10^9^	W	N
	H173	3.12 × 10^9^	0	W	5.17 × 10^7^	H	N
13^3^	H194	3.65 × 10^9^	100	H	6.50 × 10^8^	M	N
	H200	4.59 × 10^9^	60	M	8.45 × 10^8^	W	N
	H201	5.01 × 10^9^	60	W	3.77 × 10^8^	H	N

a *Based on the scores in the piglet virulence test, three strains of each serovars, one for each of the highest, medium (the median of the highest and lowest scores), and weakest, were regarded as the highly medium or weakly virulent strains of each serovars, respectively*.

*The determination of mouse virulence is based on the LD_50_ values. For serovars 4, 12, and 13, the LD_50_s of were divided into three intervals. Three strains with the lowest or the highest LD_50_s were classified as highly virulent or weakly virulent, respectively. Two strains with the moderately LD_50_s were classified as medium virulent. In the 12 G. parasuis strains of serovar 5, four strains with the lowest, moderately or the highest LD_50_s were classified as highly virulent, medium virulent or weakly virulent, respectively*.

b *“H,” “M,” “W” Represents virulent strains, medium virulent strains, and weak virulent strains in the same serovars, respectively*.

c *“N” Shows that the virulence of mice is not consistent with the virulence of piglets*.

*With reference to the incidence of piglets, determine the virulence of H200 strain and H201 strain (Table 2)*.

*The gray shades is used to distinguish the serotypes of the strains*.

## Discussion

*G. parasuis* is a common respiratory bacterium with numerous serovars. It specific culture conditions, and in particular, strictly requires NAD (factor V) for growth (1). Even under the same culture conditions, the growth rates of different *G. parasuis* strains can vary (37). For example, *G. parasuis* could not be detected when it was placed in saline or PBS at 42°C for 1 h, at 37°C for 2 h, or at 25°C for 8 h. However, the number of live bacteria was only slightly reduced when it was stored at 5°C for 8 h (38). These observations imply that the actual challenge dose of each strain differed in the piglet virulence tests in that study; The minimum challenge dose was 2.63 × 10^9^ CFU and the maximum dose was 8.61 × 10^9^ CFU. In 2004, Oliveira reported that the intraperitoneal inoculation of piglets with 10^8^-10^9^ CFU of *G. parasuis* resulted in fibrinous polyserositis, arthritis, and meningitis, whereas piglets inoculated with 10^6^–10^7^ CFU showed no symptoms (39). The results of our study using 8–9-week-old *G. parasuis*-seronegative piglets challenged with 36 *G. parasuis* strains showed that the intraperitoneal injection of *G. parasuis* successfully caused Glässer's disease, and could be used to evaluate the virulence of different strains.

According to the Kielstein-Rapp-Gabrielson serotyping scheme, serovars 5, 12, and 13 are classified as strongly virulent, and serovar 4 is classified as moderately virulent (2). However, the piglet virulence test results showed that the comprehensive virulence of the four serovars followed the order: serovar 5 > 13 > 4 > 12. Although some strains of serovars 5 and 13 caused 100% death rates among the inoculated piglets, and were therefore strongly virulent strains (strains H94 and H194), some strains did not cause a 100% death rate in the inoculated pigs, and were therefore moderately virulent or attenuated strains (strains H134 and H201). All eight strains of serovar 4 caused the death of some inoculated piglets, exhibiting virulent strains (strains H47 and H57), but it could not be identified as a moderately virulent serovar in general. The results for serovar 12 were the most divergent among the strains, insofar as strain H163 was strongly virulent, whereas strains H170, H173, and H157 caused no deaths (although disease onset was observed) and therefore display attenuated virulence. These results are inconsistent with the serological classification, suggesting that there is no correlation between the serovar and virulence of *G. parasuis*. Aragon et al. also showed that some strains of virulent serovars 4 and 10 showed no pathogenicity to NFCD pigs, whereas in contrast, some strains of avirulent serovar 7 caused severe disease in piglets (40). Yu et al. studied 10 *G. parasuis* strains and demonstrated that strains of the same serovars showed different virulence in 4-week-old York piglets (25), indicating that in *G. parasuis*, serovar does not necessarily correlate with virulence. In fact, the relationship between serovar and virulence in *G. parasuis* is not well-established because the virulence of serovars was established using only reference strains (3, 17).

Strain classification of *G. parasuis* has been widely studied serotypically and genotypically, since differentiation of non-virulent strains from virulent strains is essential for diagnosis and control of the disease. Serotyping is the most commonly used subtyping method, and is traditionally considered to be associated with virulence, but increasing evidence indicated that serovar is a poor proxy for virulence (14, 40, 41). To resolve this problem, such as genome-wide association study (GWAS), enterobacterial repetitive intergenic consensus polymerase chain reaction (ERIC-PCR) (42), multiplex PCR (mPCR) (43) and multilocus variable number of tandem repeats analysis (MLVA) (44), have been used to differentiate *G. parasuis* strains and predicte virulence-associated genes. Although several studies have identified putative virulence-associated genes, their role in pathogenesis has not been demonstrated (45–48). It is probable that the virulence of *G. parasuis* strains may have varied due to vertical and/or horizontal transfer(s) of DNA in the past 20 years. Thus, it was not exact to decide the virulence only by the Kielsteine-Rappe-Gabrielson serotyping scheme. The animal challenge and gene virulence detection would be useful supplements to the Kielsteine-Rappe-Gabrielson serotyping scheme, which evaluates bacterial virulence of *G. parasuis*.

We used BALB/c mice as an alternative model of *G. parasuis* infection to test the virulence of the four most domestically common serovars of the bacterium. The results showed that the LD_50_s of serovar 13 were between 1.75 × 10^8^ and 8.45 × 10^8^ CFU, whereas the LD_50_s of the other three serovars varied more widely. These results are consistent with those of many scholars, who showed that the use of intraperitoneal injection of *G. parasuis* causes death in BALB/c mice (49, 50); However, these scholars didn't compare the virulence of these strains in piglets. We speculated that different infection routes would produce different virulence results. Gao et al. (51) studied various inoculation routes of *G. parasuis* in guinea pigs and demonstrated that intraperitoneal or intrapulmonary routes were more sensitive than intranasal, intramuscular, and subcutaneous routes (52, 53). In addition, considering the experimental operation, abdominal infection is simpler and easier to operate than other infection methods. To compare the virulence of *G. parasuis* in mice and piglets in this study, the intraperitoneal injection was used in mice and piglets. The results of our virulence tests in BALB/c mice were not consistent with those in piglets. The mouse virulence test results showed that serovar 13 displayed the strongest virulence, followed by serovars 4, 12, and 5, and that the virulence of serovar 5 was significantly weaker than that of serovar 13 (*P* < 0.05). However, the piglet virulence test results showed that serovar 5 was most virulent, followed by serovars 13, 4, and 12, and that the virulence of serovars 5 and 13 did not differ significantly (*P* > 0.05). Our results also showed that even the same strain of *G. parasuis* could display different virulence in mice and piglets. From an anatomical pathology perspective, we found that the BALB/c mice predominantly died from internal organ bleeding. Because pigs are a natural host of *G. parasuis*, the virulence test results for piglets better reflect the virulence of these common strains, our results also indicate that BALB/c mice are inadequate as an alternative model of *G. parasuis* infection.

## Conclusion

In this study, the virulence of the commonest serovars (4, 5, 12, and 13) of *G. parasuis* in China (a total of 36 strains) was compared in BALB/c mice and piglets. Three conclusions can be drawn, from our results: (1) in piglets, the virulence of serovar 5 was the strongest, followed by that of serovars 13, 4, and 12; (2) both virulent and avirulent strains were present in all the serovars and there was no correlation between serovar and virulence; and (3) although *G. parasuis* caused death in BALB/c mice, the virulence test results of mice were not consistent with those of piglets, indicating that BALB/c mice are inadequate as an alternative model of *G. parasuis* infection.

## Data Availability Statement

The raw data supporting the conclusions of this article will be made available by the authors, without undue reservation.

## Ethics Statement

All procedures performed in studies involving animals were in accordance with the Animal Experiment Committee of Henan University of Science and Technology (No. 20180719009). The samples were collected and handled in accordance with the good animal practices required by the Animal Ethics Procedures and Guidelines of the People's Republic of China.

## Author Contributions

ZZ designed the study. BQ performed experiments. BQ, FL, KC, WD, YW, YX, HW, and KD performed data analysis. BQ and ZZ wrote the draft and revised the manuscript. All authors have read and approved the manuscript.

## Conflict of Interest

The authors declare that the research was conducted in the absence of any commercial or financial relationships that could be construed as a potential conflict of interest.

## Supplementary Material

Supplementary Figure 1 Symptoms of mice infected with *G. parasuis*. **(A–F)** pleural effusion, peritoneal fibrous exudate; **(G)** liver hemorrhage, congestion and necrosis; **(H)** pulmonary congestion and hemorrhage; **(I)** heart congestion.

Supplementary Figure 2 The vitro growth curve for 36 isolates in 6–14 h. A single colony of *G. parasuis* was picked and cultured in TSB medium containing newborn calf serum (10%) and NAD (10 μg/mL), with shaking at 180 rpm at 37°C for 12–16 h. The culture solution was used as the stock solution and diluted 1:100, transferred to TSB medium, and cultured with shaking at 180 rpm at 37°C. From 6 to 14 h, the number of living bacteria in the bacterial liquid was recorded every 2 h.

## Abbreviations

CDCD: Cesarean-derived, colostrum-deprived
NFCD: Naturally-arrowed, artificially-reared
NAD: Nicotinamide adenine dinucleotide
MAT: Microplate agglutination test
LD_50_: Median lethal dose
ANOVA: Analysis of variance
TSB: Tryptic soy broth
TSA: Tryptic soy agar
CFU: Colony–forming units.
