# Supplementary figures and images for: Comparison of the Glaesserella parasuis Virulence in Mice and Piglets
Source: Front Vet Sci. 2021 Jun 24;8:659244. doi: 10.3389/fvets.2021.659244 (PMC8265781; doi:10.3389/fvets.2021.659244)

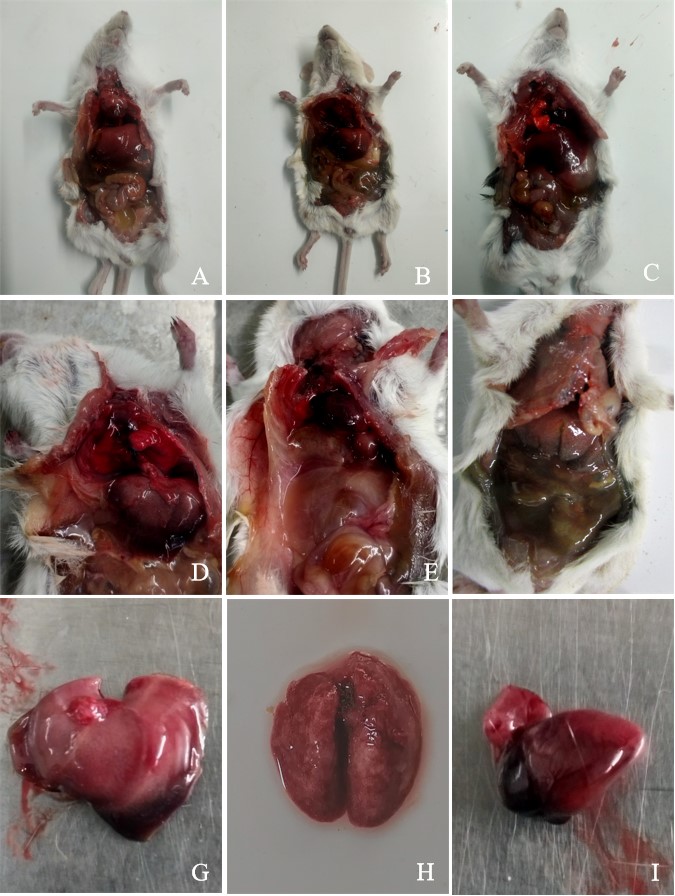

Supplement: Supplementary Figure 1 — Symptoms of mice infected with G. parasuis. (A–F) pleural effusion, peritoneal fibrous exudate; (G) liver hemorrhage, congestion and necrosis; (H) pulmonary congestion and hemorrhage; (I) heart congestion. [file Image_1.JPEG]

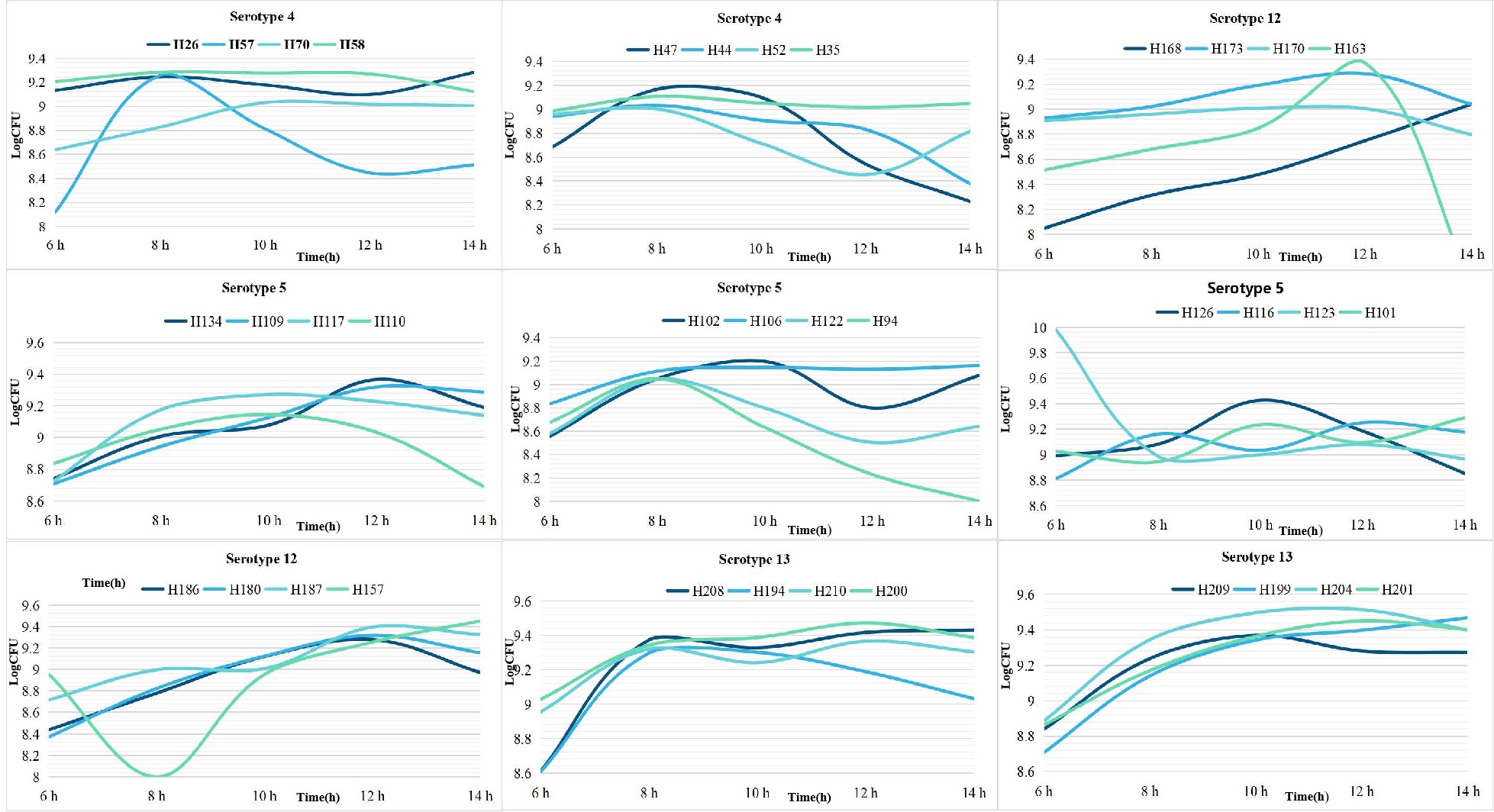

Supplement: Supplementary Figure 2 — The vitro growth curve for 36 isolates in 6–14 h. A single colony of G. parasuis was picked and cultured in TSB medium containing newborn calf serum (10%) and NAD (10 μg/mL), with shaking at 180 rpm at 37°C for 12–16 h. The culture solution was used as the stock solution and diluted 1:100, transferred to TSB medium, and cultured with shaking at 180 rpm at 37°C. From 6 to 14 h, the number of living bacteria in the bacterial liquid was recorded every 2 h. [file Image_2.JPEG]
